# 5-SPICE: the application of an original framework for community health worker program design, quality improvement and research agenda setting

**DOI:** 10.3402/gha.v6i0.19658

**Published:** 2013-04-03

**Authors:** Daniel Palazuelos, Kyla Ellis, Dana DaEun Im, Matthew Peckarsky, Dan Schwarz, Didi Bertrand Farmer, Ranu Dhillon, Ari Johnson, Claudia Orihuela, Jill Hackett, Junior Bazile, Leslie Berman, Madeleine Ballard, Raj Panjabi, Ralph Ternier, Sam Slavin, Scott Lee, Steve Selinsky, Carole Diane Mitnick

**Affiliations:** 1 Partners In Health, Boston, MA, USA; 2 Division of Global Health Equity, Department of Medicine, Brigham and Women's Hospital, Boston, MA, USA; 3 Department of Global Health and Social Medicine, Harvard Medical School, Boston, MA, USA; 4 Harvard Medical School, Boston, MA, USA; 5 Nyaya Health, Boston, MA, USA; 6 Project Muso, Boston, MA, USA; 7 Tiyatien Health, Boston, MA, USA; 8 Harvard Business School, Boston, MA, USA

**Keywords:** community health workers, quality improvement, research, conceptual framework, case study

## Abstract

**Introduction:**

Despite decades of experience with community health workers (CHWs) in a wide variety of global health projects, there is no established conceptual framework that structures how implementers and researchers can understand, study and improve their respective programs based on lessons learned by other CHW programs.

**Objective:**

To apply an original, non-linear framework and case study method, 5-SPICE, to multiple sister projects of a large, international non-governmental organization (NGO), and other CHW projects.

**Design:**

Engaging a large group of implementers, researchers and the best available literature, the 5-SPICE framework was refined and then applied to a selection of CHW programs. Insights gleaned from the case study method were summarized in a tabular format named the ‘5×5-SPICE chart’. This format graphically lists the ways in which essential CHW program elements interact, both positively and negatively, in the implementation field.

**Results:**

The 5×5-SPICE charts reveal a variety of insights that come from a more complex understanding of how essential CHW projects interact and influence each other in their unique context. Some have been well described in the literature previously, while others are exclusive to this article. An analysis of how best to compensate CHWs is also offered as an example of the type of insights that this method may yield.

**Conclusions:**

The 5-SPICE framework is a novel instrument that can be used to guide discussions about CHW projects. Insights from this process can help guide quality improvement efforts, or be used as hypothesis that will form the basis of a program's research agenda. Recent experience with research protocols embedded into successfully implemented projects demonstrates how such hypothesis can be rigorously tested.

Community health workers (CHWs) have worked in clinical and public health projects for over half a century, delivering services to marginalized populations in resource-limited settings, decentralizing care down to the community level, and fundamentally linking communities to the formal health system (1–3). Despite these many decades of experience, there is little consensus on how an ideal CHW project should be structured when developed, and how challenges or opportunities should be negotiated once in practice. A number of useful ‘tool kits’ to improve programs are available (4, 5); however, an adaptable framework to analyze and derive insights from the full range of CHW programs does not yet exist. At best, this framework would need to be understandable to all partners – from the CHWs themselves to project leadership – yet robust enough to support advanced study of the complex unanswered questions in CHW science. Partners In Health (PIH), a Boston-based nongovernmental organization (NGO), offers a new conceptual model for how to approach, construct, and sustain high-performing CHW programs. Culled from over 25 years of experience on almost every continent, we believe that this model will help to strengthen and expand the current conversation about CHWs.

## The 5-SPICE framework case study method

Traditional CHW conceptual models and tool kits focus on program inputs, outcomes, and impacts (1, 3). These cause-and-effect elements are often positioned in a linear fashion. However, most global health delivery (GHD) projects are far more complex, multivariate, and non-linear in reality. Any CHW framework will need to reflect the myriad *interactions* among program elements to be useful.

We offer the 5-SPICE framework as a response to this need. While a more in-depth explanation of the framework's structure and origins will be described in a separate publication (6), what we offer in this article is an outline of the core tenets that were integral to the framework's implementation. The term ‘5-SPICE’ is borrowed from Chinese cooking, in which five spices are used by chefs to achieve a balance of flavors in a dish. Analogously, the 5-SPICE framework utilizes five elements, spelled out in an acronym, which can serve as essential ingredients whose components and interactions should be considered in unison when structuring or analyzing a CHW program:

*S*upervision (including management plans and structures)

*P*artners (especially ownership and stewardship by national programs)

*I*ncentives (which are a key part of the larger theme of motivation and performance)

*C*hoice (both how CHWs are recruited to work, screened, and selected, and why they choose to take the job)

*E*ducation (including what CHWs bring to their job, and how they are trained)

This metaphor was selected because both the theory and practice of cooking represents a common shared experience with which most groups will be familiar; it is an essential part of the human experience, but it can also be a highly complex practice when pursued in real-world contexts. Authoring, managing, and improving CHW programs in diverse global health contexts can metaphorically be seen as sharing important similarities with the practice of cooking; although interactions may follow intricate and chaotic processes, past experiences can be used to elucidate patterns. In turn, these linkages and pathways can guide future decision-making. The ultimate goal when using this model is to first understand the five elements and their interactions to then design, manage, and/or help evolve effective programs. Our core hypothesis is that maintaining this conceptual ‘balancing act’ between elements may improve how GHD implementers working with CHW programs can artfully craft, understand, and sustain quality interventions in challenging and ever-changing, global health environments.

**Fig. 1:**
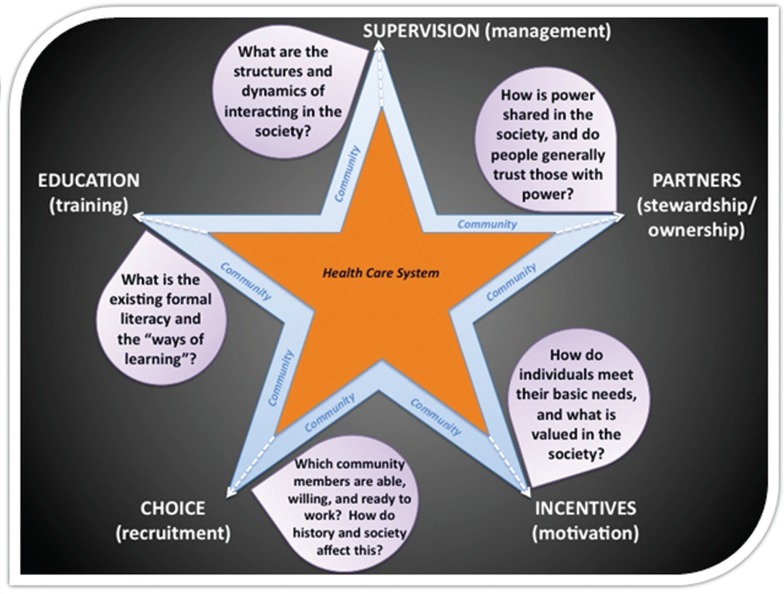
The 5-SPICE framework graphically represented.

As seen in Fig. 1, each point of the star represents one of the elements for structuring and managing a CHW program. Each is uniquely important, but when combined, their whole is greater than the sum of their parts. As part of a unified structure, the positioning of each element will by definition affect all the others. There are actually two embedded stars, each representing the unique contributions of the community with which the intervention is being implemented (the blue star) and the health care delivery system (the orange star). This overlapping star graphic is a visual reminder that each element should always be conceptualized in how it simultaneously interacts within and between both the community and the health care system.

In addition, the economic, sociocultural, historical, and political terrain of the local context influences how each input will work in that unique setting; what we consider to be the most essential questions raised by these ‘contextualizing considerations’ are represented by the questions or ‘anchoring points’ in the purple bubbles adjacent to each input.

## Methods

PIH is a Boston-based NGO that aims to treat the sickest and poorest patients on the planet with the highest standards of care available. Partnering with community-based organizations and CHWs has long been one of the most important strategies by which PIH has achieved its mission (7–10). After more than 25 years in operation, ‘the PIH model’ has proposed five principles that are essential to providing community-based care with an equity agenda: 1) access to primary care builds trust among community members in the health care system; 2) free health care and education eliminates many of the barriers that prevent access; 3) community partnerships that are based on mutual respect allow communities to let their needs be known; 4) addressing basic social and economic needs helps to fight the root of disease in impoverished communities; and 5) serving the poor through the public sector is the best mechanism by which the right to health can be conferred (11).

Starting in the summer of 2010, PIH began investing in a CHW cross-site quality improvement task force in an effort to encourage collaborations between, and best-practice sharing across, the many country projects currently associated with the NGO. After convening the task force as a consortium of site-specific representatives, the group was able to undertake a number of activities that had the ultimate goal of better understanding the commonalities and differences of our various participating projects. These tasks included holding monthly teleconferencing meetings, generating cross-site statistics via the application of an original data-gathering instrument called ‘the PIH CHW rich grids’, sharing internal materials through an Internet-based cloud service, and convening all the task force representatives at an annual PIH CHW summit. The most active members of the task force have also represented PIH during collaborations with other NGOs, such as assisting in the Earth Institute's ‘One Million Community Health Workers Technical Task Force Report’, and during national and international CHW meetings, such as the United States Agency for International Development (USAID) CHW summit held in Washington, DC, in 2012 (12). Through these efforts, and especially after the PIH and USAID summits of 2012, the authors of this article formulated the 5-SPICE framework. We utilized expert opinion, personal experience, and the best available evidence in a variety of disciplines when considering what elements should be included as essential components. The first author, also the CHW task force director, further refined the model after consulting a variety of other PIH collaborators, including those from the business, management, education, public health, and medical sectors (see Acknowledgements).

**Chart 1:**
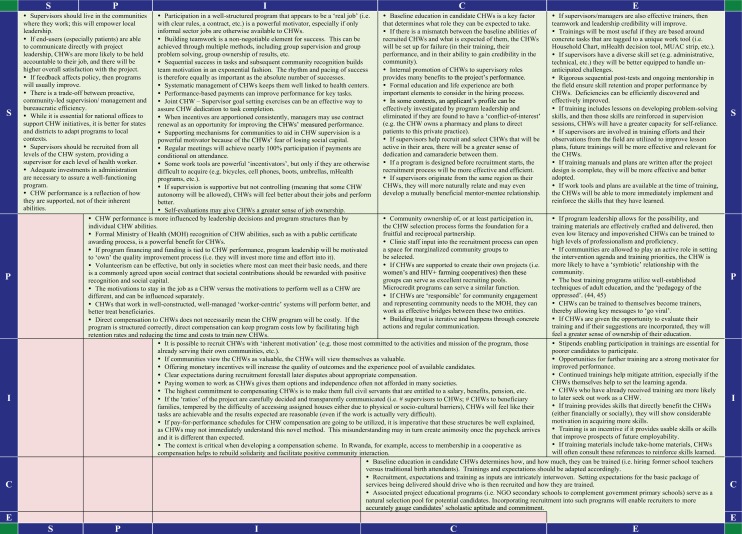
5×5 SPICE chart-positive interactions.

**Chart 2:**
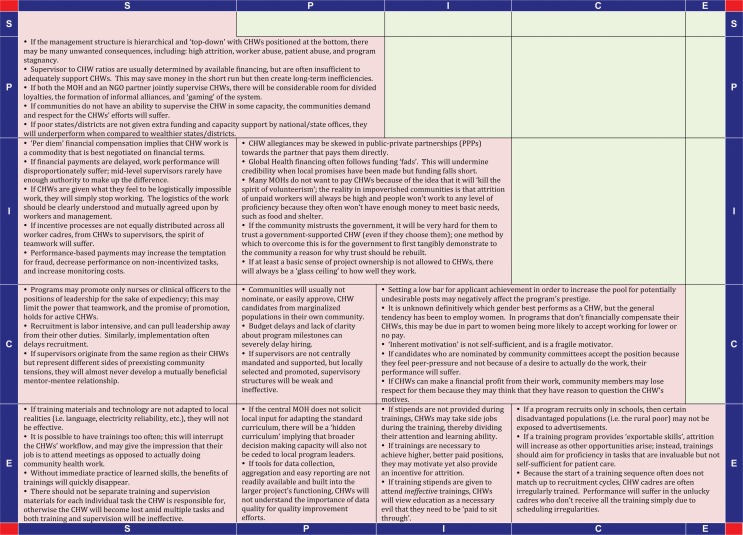
5×5 SPICE chart-negative interactions.

This manuscript represents the effort of the PIH CHW task force to apply the framework as a trial run of a novel case study method. This was achieved primarily by the PIH CHW task force representatives who each utilized the model to provide insights about their projects from their unique vantage points in the field. The process of utilizing the model to analyze each site, what we call ‘the 5-SPICE case study method’, included two key steps: first developing a narrative about how each of the 5-SPICE elements interacted within the program from its inception to its current day iteration, and then completing a ‘5×5-SPICE chart’, in which the program representatives located insights at the intersection of two interacting elements. They were asked to consider their program's initial design and structure, the mid-course corrections needed once that structure was first being implemented, and the resultant successes and/or failures. We compared these insights, identified common themes, and then compiled them into two master ‘5×5-SPICE charts’ (see Charts 1 and 2). The first chart lists how different 5-SPICE elements interacted in positive or beneficial ways; the second chart lists how different elements interacted in negative or detrimental ways.

The projects included in this exercise comprised: 1) PIH core projects; 2) smaller PIH projects; 3) projects associated with PIH, which form an implementation network aiming to adapt core elements of the PIH model, yet maintain their own operations; and 4) other highly esteemed CHW projects outside of PIH. The PIH core projects are those that receive direct funding and have a mandate to implement the core elements of ‘the PIH model’ mentioned above, including Haiti, Rwanda, Malawi, and Lesotho. These four countries are PIH's comprehensive primary care sites, where the organization partners with multiple government-run health centers and district hospitals to directly provide care to patients. Other PIH projects that also follow the PIH model but on a smaller scale include those from Mexico, USA, and Peru. The participating associated projects include those in Liberia, Mali, and Nepal. By inviting all these groups to participate, we widened the variety of programs contributing to this exercise. This we found, in turn, increased the richness of insights culled from the process.

Finally, to assess the model's adaptability, the team sought to apply the model to cases beyond its network. To do this, a Harvard medical student used the case study method to analyze a number of other CHW projects that have been widely described in the literature, including BRAC in Bangladesh (13–24), and the Health Extension Worker program in Ethiopia (25–31). Discussions that the task force had with researchers active in national CHW programs being implemented in Zambia and India (the ASHA project) also influenced the insights reported.

These non-PIH CHW projects were selected because they are generally held in high regard and because there is a wealth of information published on their inner workings. Since this was the initial pilot application of a novel iteratively built framework, the selection of projects was not exhaustive and did not utilize any rigorous process for inclusion or exclusion.

## Results

**Fig.2:**
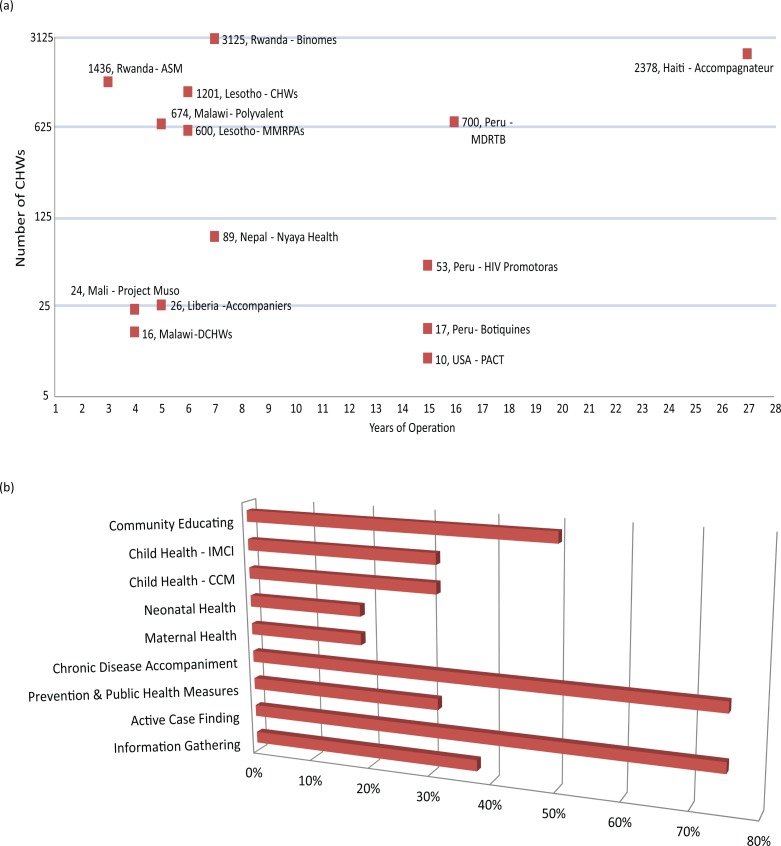

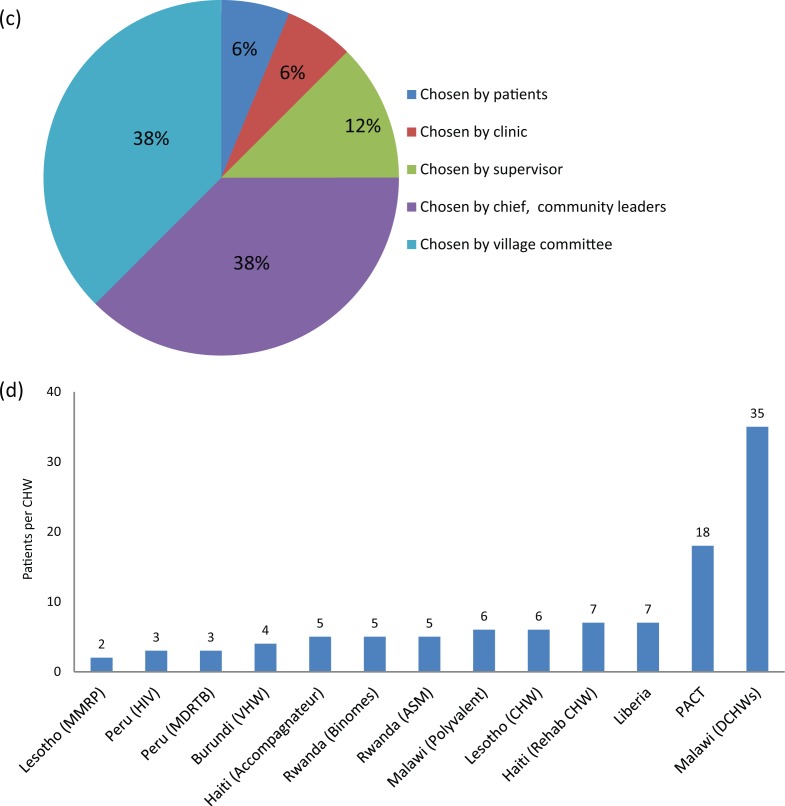
Partners In Health (PIH) CHW Project characteristics (from the ‘Rich grids’). (a) Number of CHWs vs. Years in operation for various PIH CHW projects. (b) Distribution of CHW package of service across PIH programs. (c) CHW selection and recruitment at PIH programs. (d) Patient load per CHW by PIH program. *Note*: Data for PIH-Mexico are not yet available due to ongoing changes in this new program's implementation.

Figure 2a–d represents key statistics generated from the ‘PIH rich grids’ results. Figure 2a shows how the projects represented in this article group up around a few size trends based on their age: while one of the largest projects at PIH is by far the oldest (Haiti), there are a number of newer ‘scale-up’ projects that also boast large cadres of CHWs. The smaller PIH projects and associated projects span a wider range of size per age, but in general are much smaller. Figure 2b shows that the two most common activities in which CHWs at PIH engage are active case finding and chronic disease accompaniment. Community education is also a common activity. The existence of cadres of CHWs who perform community case management (CCM) or integrated management of childhood illnesses (IMCI) activities is less common, though present. Figure 2c shows that the vast majority of CHWs are chosen by the chief or by a village committee (38% and 38%, respectively). Figure 2d shows that aside from two outliers that are responding to context-specific requirements, the majority of programs surveyed maintain a low CHW to beneficiary ratio.

In addition to those represented in these figures, the ‘rich grids’ generated other statistics of interest. For example, in terms of literacy requirements, 87% of CHW programs surveyed require literacy as the basic educational prerequisite of the program and 19% require a secondary school education for employment. In terms of payment structure, 50% pay their CHWs on a monthly stipend or salary schedule, 30% receive cash for tasks, and 20% receive non-monetary compensation and incentives, including membership in cooperatives. The initial training requirements vary by site and program, and range from 6 hours of classroom education in specific Malawian programs to a 12-week program in Haiti. Excluding this 12-week program, the average initial training is 48 hours, or approximately 5 days. Also, 81% of the programs surveyed have continuing education programs, ranging from monthly to bi-yearly.

Charts 1 and 2 display the insights provided by the participating CHW projects that contributed a program narrative and a 5×5-SPICE chart (positive and negative interactions, respectively). These are summarized as short statements that posit the ways in which various 5-SPICE elements interact and influence the programs’ implementation. Some of these statements have been well described in the literature previously, while others are exclusive to this article.

While there are too many statements to discuss each in detail here, one trend bears mention as it illustrates the power of this framework to elucidate unique insights: the question of how to best incentivize CHWs. Mirroring the core argument of the 5-SPICE framework that key elements are best understood as they relate to each other instead of in isolation, the statements in the 5×5-SPICE charts reflect the need for a more contextualized approach to selecting the best strategy for CHW motivation, one that balances goals and ethics with inputs and expectations.

For example, two statements from the 5×5-SPICE chart (positive interactions) state that while ‘it is possible to recruit CHWs with “inherent motivation”,’ it has also been observed that ‘offering monetary incentives will increase the quality of outcomes and the experience pool of available candidates’. This suggests that different CHW cadres, recruited, trained, and supervised differently, should be asked to perform different tasks and produce different results. If the task at hand is technically simple and allows for some work plan flexibility, but also requires trust and an enhanced social standing to be implemented well (such as community education around the benefits of family planning), there could be an opportunity for enrolling CHWs in multiple areas on a non-salaried basis. Multiple statements in the 5×5-SPICE charts also suggest that the specialized training and work tools (i.e. flipcharts, etc.) that these CHWs may receive, and the enhanced social capital generated from the activity, will themselves be a form of motivation for the CHW. Another statement from the same chart puts forth a hypothesis in support of this sentiment: ‘volunteerism can be effective, but only in societies where most can meet their basic needs, and there is a commonly agreed upon social contract that societal contributions should be rewarded with positive recognition and social capital’.

On the other hand, many statements suggest that if a task is technically difficult, requires a more defined work plan, and demands that administrative tasks such as record keeping be of high quality, (such as providing curative care for childhood illnesses, HIV, tuberculosis or non-communicable diseases), then this CHW cadre would benefit from more substantial investments, such as a careful selection process, a specialized education, and adequate compensation. Another statement asserts: ‘direct compensation to CHWs does not necessarily mean the CHW program will be costly. If the program is structured correctly, direct compensation can keep program costs low by facilitating high retention rates and reducing the time and costs to train new CHWs’. Compensation for these high-value CHWs can come in many forms, from salaries with benefits to access to capital investments through a CHW led cooperative. As the same chart explains, ‘the context is critical when developing a compensation scheme. In Rwanda, for example, access to membership in a cooperative helps to rebuild solidarity and facilitate positive community interaction’. Another statement from the ‘Negative Interactions’ chart warns, however, ‘if CHWs can make a financial profit from their work, community members may lose respect for them because they may think that they have reason to question the CHW's motives’.

The core message being woven from these statements is not only which strategy is better, but also which strategy will be relatively better, more reasonable, and more feasible for that context in order to reliably produce the expected results.

## Discussion

This article illustrates how PIH and its academic partners developed an original CHW conceptual framework, called 5-SPICE, and then applied it as a case study methodology to its own, and other, CHW projects. The selection of CHW programs examined was diverse. The ‘rich grid’ results demonstrate how the projects participating in these exercises span a wide variety of structures and experiences: some are decades old and others are only now starting; some are large and employ thousands of CHWs, others are small and maintain only a handful of active but highly trained and supported CHWs. While there is considerable variety in almost every variable, a key trend is that although PIH's primary experience with CHWs has been with Accompagnateurs (or patient support CHWs that improve adherence and clinical outcomes for HIV and TB), PIH is also expanding in both the scale and scope of its work.

This expansion and diversification in PIH's operations was reflected in the richness of insights compiled in the 5×5-SPICE charts. One in particular that was examined in detail was the question about how best to compensate a CHW. This topic is critical for many reasons: how it is answered will determine what resources will be necessary for a program's implementation strategy, its capacity for scale-up, and its long-term sustainability (32–34). The various options available to program architects can be positioned along a spectrum ranging from pure volunteerism on one side and salaried, if not civil servant, workers on the other (35). Between these options, there are a variety of choices available that will motivate useful CHW behavior based on intrinsic factors (e.g. working due to sense of moral or community duty) or extrinsic factors (e.g. working in order to secure a livelihood, maybe even a way out of poverty) (36). The 5×5-SPICE analysis suggests that diametrically extreme viewpoints may actually be too simplified to be useful. The truly ‘best-practice’ by which to motivate CHW behavior will be relative to the task being asked of the CHW, how the CHW is selected to do the job, the expected results of that task, and how the community will view that motivation strategy. Volunteerism is an option when limited to relatively straightforward initiatives that do not require a large start up investment and allow the CHW some flexibility in how they approach the task. They can also be bolstered if there is a strong societal priority for valuing their contributions. Our analysis suggests that volunteerism, however, has very real limitations. If impoverished volunteer CHWs cannot live off of their activities, their ability to do the job will be very fragile; competing challenges that may arise will usually be prioritized before these tasks, especially if the CHW is female and is also responsible for a myriad of other unpaid tasks. This is likely the best explanation for a well-known and vexing phenomenon: unpaid workers have the highest rates of attrition (37).

For the cadres of CHWs that are asked to perform more time-intensive and quality-sensitive tasks, a more concerted investment in their recruitment and retention is prudent. After applying this investment, it would be shortsighted to employ only volunteers because any attrition will represent a substantial loss of the original start-up costs. Following this logic, any continued effort to engage in strategies that prevent attrition and improve quality, such as continued team-building exercises, a clear scope-of-work plan with adequate supervision, and community recognition mechanisms, will only reinforce the value generated by the project and the investment. When compensating CHWs financially, however, care should be given to choose the mechanism that is best for that context and will not generate jealousies or encourage perverse incentives.

It is this type of analysis that we believe the 5-SPICE case study method to be capable of yielding. Nevertheless, we also envision a number of other applications for the framework. First, this framework can be utilized when designing a project. It will help when considering the available inputs via the five elements proposed, and will assist in judging the local sociocultural terrain via the ‘anchoring points’. Aiming for balance between these elements will allow for a richer analysis of where reinforcements will be necessary to compensate for inputs that simply are less strong in that context. The second application will be when a project that is already developed needs to be improved. Each input can be mapped out, and through the creation of 5×5-SPICE charts, strengths and weaknesses can be discovered. The framework provides a concrete method by which to get a ‘bird's-eye view’ of a program before beginning an iterative analysis of how a quality improvement strategy may be pursued. The 5×5-SPICE charts are an exercise that encourages program staff to think of their programs as holistic and intricately interwoven. The simplicity of the framework, and the universality of the cooking metaphor, will hopefully allow a variety of voices and perspectives into the conversation; it has been our experience that by including in the analysis process all groups affected by the program, from staff to end-user beneficiaries, indispensable insights will arise.

Another way in which the insights gleaned from the application of the 5-SPICE framework can be utilized is to help set a research agenda. For example, each statement recorded in the 5×5-SPICE charts can be seen as hypotheses that may inform future research priorities. The best way to later test these hypotheses is by studying CHW programs in their context. For implementing NGOs such as PIH, the most economical and programmatically feasible evaluations consist of adequacy assessments, or pre–post intervention studies. More rigorous quasi-experimental designs involving control groups (plausibility assessments) may be possible if the organization can select a subset of its CHWs to receive an intervention and study its effect relative to a comparison group. Recently published experiences have demonstrated that it is becoming more feasible to conduct such nested randomized experiments (probability assessments) within operating global health programs (38, 39). If there are concerns about withholding an intervention from a comparison group, a ‘stepped wedge’ study design can be pursued instead (40).

The experience in Zambia, for example, has important lessons for the direction that new programs can take. The Zambian CHW program currently being implemented has within its design a number of experiments that will provide some of the most definitive conclusions available to date on CHW program design. For example, an imbedded recruitment experiment randomized two different recruitment strategies, one aiming to recruit individuals who are more intrinsically motivated (e.g. have a strong desire to serve their communities) but might have less technical capacity, and another aiming to recruit those who are more extrinsically motivated (e.g. have a strong desire to meet performance targets and earn personal benefits) but might be more likely to leave the post if more attractive opportunities arise. The researchers will be able to observe their performance over time and draw further conclusions about the long-run impact of selecting for different types of CHWs, on measures such as retention, quality of care, and responsiveness to community needs. Other embedded experiments are also studying different training incentives, and the effects of goal-setting on CHW performance (41, 42). This is similar to other studies, also conducted in Zambia, that assessed the effect of different incentive structures on hairdressers who promote condom use by randomly assigning them to varying payment schemes and then monitoring the effect on condom sales (43). Results from studies such as this – coupled with thorough documentation on intervention costs – can provide valuable evidence to policymakers interested in the scale-up of different insights provided by the 5-SPICE method presented in this paper.

The more global health researchers and implementers utilize the 5-SPICE framework and describe their experiences within its logic structure, the more useful it will become for future users. It is still unclear how the five elements of this framework will interact in the full variety of global health contexts available for analysis, but with time and experience, we will be able to build a clearer understanding of trends and commonalities. Biochemistry provides a just illustration for how this science may be built: the Krebs cycle, for example, is a complex set of pathways that were each discovered sequentially but then were unified thematically to conceptually form a beautifully simple process that explains some of the most critical biochemical processes in the body. Like a puzzle, the discovery of each smaller pathway contributed a piece to the final solution. What guided this process was a common pursuit and mutually agreed upon conceptual framework. Similarly, the 5-SPICE method may allow us to consider all the moving elements, the particular causative pathways that coalesce to make a CHW program successful or not in its own unique context.

Simply put, the 5-SPICE framework begins to provide us with a common language that may structure and enrich future discussions. These insights can improve the foundation upon which programs are built, refine quality improvement processes within existing programs, and help generate a series of hypotheses that can be used to formulate a concerted research agenda. We believe this will allow various stakeholders to better understand the part they play, and how their inputs have both great influence and important limitations. Nevertheless, it would be misguided to say that because this framework argues for a more contextualized approach, all GHD strategies and objectives are only relative and therefore equally as valid. Improved patient and population health with an equity agenda is the life energy that drives the programs represented in this analysis, and should be the moral compass that guides us through the development of our new CHW science.

## Limitations

The 5-SPICE framework is imperfect; as with any tool, its application is not universal and it will not be difficult to find cases that are beyond its scope and reach. Any attempt to standardize the way we think of programs will by definition limit outliers. This could have the unwanted effect of inhibiting innovation and individual voices. In addition, the components within the framework contain concepts already well-known to the business/management literature. Yet beyond a repackaging of existing ideas, we believe that this framework will allow global health implementers a common language and unique methodology that mirrors the complex realities encountered on-site. The 5-SPICE framework is still a work in progress. We invite dialogue that will help improve the tool's applicability and universality.
